# Massive Superinfected Thrombosis of Dual-Frame Transcatheter Mitral Valve Replacement

**DOI:** 10.1016/j.jaccas.2024.102456

**Published:** 2024-08-21

**Authors:** Saed Alnaimat, Srijana Maharjan, Georgios Lygouris

**Affiliations:** a Department of Cardiology, Allegheny General Hospital, Pittsburgh, Pennsylvania, USA; b Department of Internal Medicine, Allegheny General Hospital, Pittsburgh, Pennsylvania, USA

**Keywords:** echocardiography, endocarditis, imaging, mitral valve replacement, rheumatic heart disease, risk factor, thrombosis

## Abstract

Dual-frame transcatheter mitral valve replacement has emerged over the last decade as an alternative to mitral valve surgery in patients with severe mitral regurgitation, especially in the presence of prohibitive operative risk. This paper presents a case of massive superinfected thrombosis of the Tendyne valve tether along with its risk factors, diagnosis, and management.

## Graphical Abstract

**Figure:**
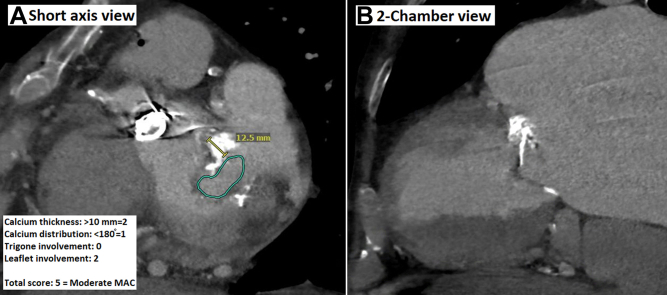

Severe symptomatic mitral regurgitation (MR), whether primary or secondary, is associated with high mortality and morbidity. Although medical therapy can be attempted, it is not effective for primary MR and occasionally effective for secondary MR. Therefore, patients commonly end up requiring surgical repair or replacement. Unfortunately, up to 50% of patients are denied surgery due to prohibitive operative risk or difficult anatomy.^1^^,^^2^ Transcatheter mitral valve replacement (TMVR) has emerged over the last decade as an alternative option for these patients.

**Learning Objectives**

• To illustrate the role of multimodality imaging in the diagnosis and follow-up of TMVR-related endocarditis.
• To emphasize the need for proper medical management of TMVR, including postoperative anticoagulation.

One of the most researched TMVR devices is a dual-frame, self-expanding bioprosthesis deployed via a transapical approach and tethered to an apical pad (referred to hereafter as dual-frame TMVR). Herein, we present a patient who underwent implantation with such a dual-frame TMVR device, later presenting with a very large infected thrombus on the valve anchor.

## History of Presentation

A 77-year-old man presented with worsening dyspnea on exertion and NYHA functional class III symptoms.

Physical examination revealed a blood pressure of 116/68 mm Hg, a heart rate of 73 beats/min, and an oxygen saturation of 97% on room air. Jugular venous pressure was slightly elevated at 10 cm H_2_O. Cardiac auscultation showed an irregularly irregular rhythm, a soft S_1_, a mechanical S_2_, and pansystolic and diastolic rumbling murmurs over the cardiac apex.

## Past Medical History

The patient has a history of rheumatic heart disease, status post ball-in-cage mechanical aortic valve replacement, mitral stenosis (MS), and permanent atrial fibrillation, for which he is on warfarin.

## Differential Diagnosis

The differential diagnosis is broad and includes heart failure exacerbation, valvular dysfunction, and pneumonia.

## Investigations

**Figure 1:**
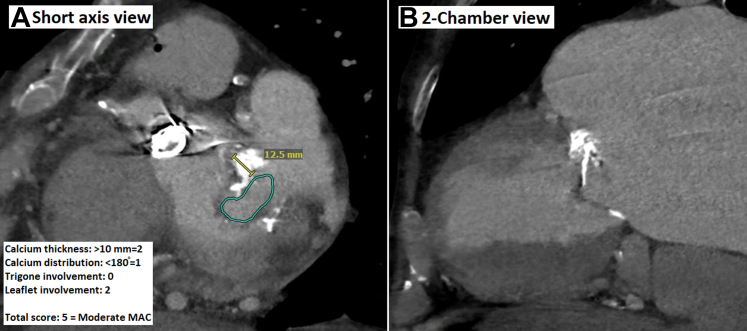
Computed Tomography of the Chest Showing Moderate MAC (A) Short-axis view. (B) Two-chamber view. MAC = mitral annular calcification.

A transthoracic echocardiogram (TTE) revealed a left ventricular ejection fraction of 52%, with a well-seated and functioning mechanical aortic valve replacement, moderate MR, and severe MS. A chest computed tomography showed moderate mitral annular calcification (Figure 1). A transesophageal echocardiogram confirmed moderate MR and severe calcific MS, with a mitral valve area of 1.38 cm^2^ by 3-dimensional reconstruction. The mean mitral gradient was 9 mm Hg at a heart rate of 70 beats/min.

## Management

The patient was subsequently enrolled in the SUMMIT (Clinical Trial to Evaluate the Safety and Effectiveness of Using the Tendyne Transcatheter Mitral Valve System for the Treatment of Symptomatic Mitral Regurgitation) trial and underwent implantation with a dual-frame TMVR device. Warfarin was resumed at 6 mg daily with a target international normalized ratio (INR) of 2.5 to 3.5. Unfortunately, his course was complicated by gastrointestinal bleeding 1 month postoperatively. He underwent multiple endoscopic procedures without a source of bleeding being found. Warfarin was briefly held and then resumed with a lower target INR of 2.5 to 3.

**Figure 2:**
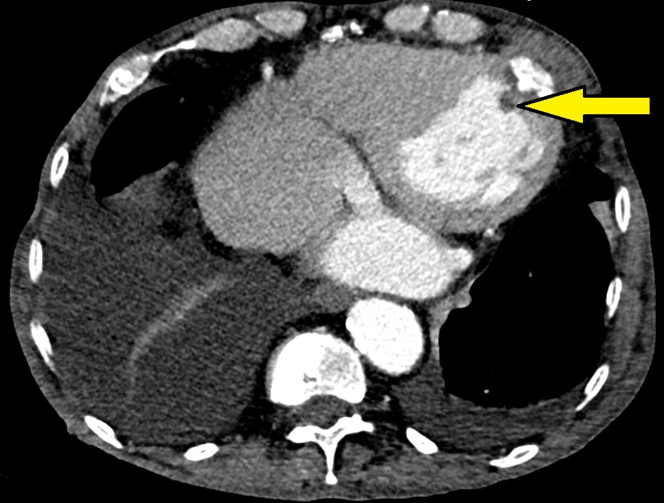
Computed Tomography Angiogram of the Chest Computed tomography angiogram of the chest showing a thrombus attached to the apical end of the dual-frame transcatheter mitral valve replacement tether (yellow arrow).

A computed tomography angiogram of the chest obtained 5 months postoperatively was suggestive of a 12-mm thrombus adherent to the apical end of the tether (Figure 2). This was managed medically by increasing the target INR range to 3 to 3.5.

**Figure 3:**
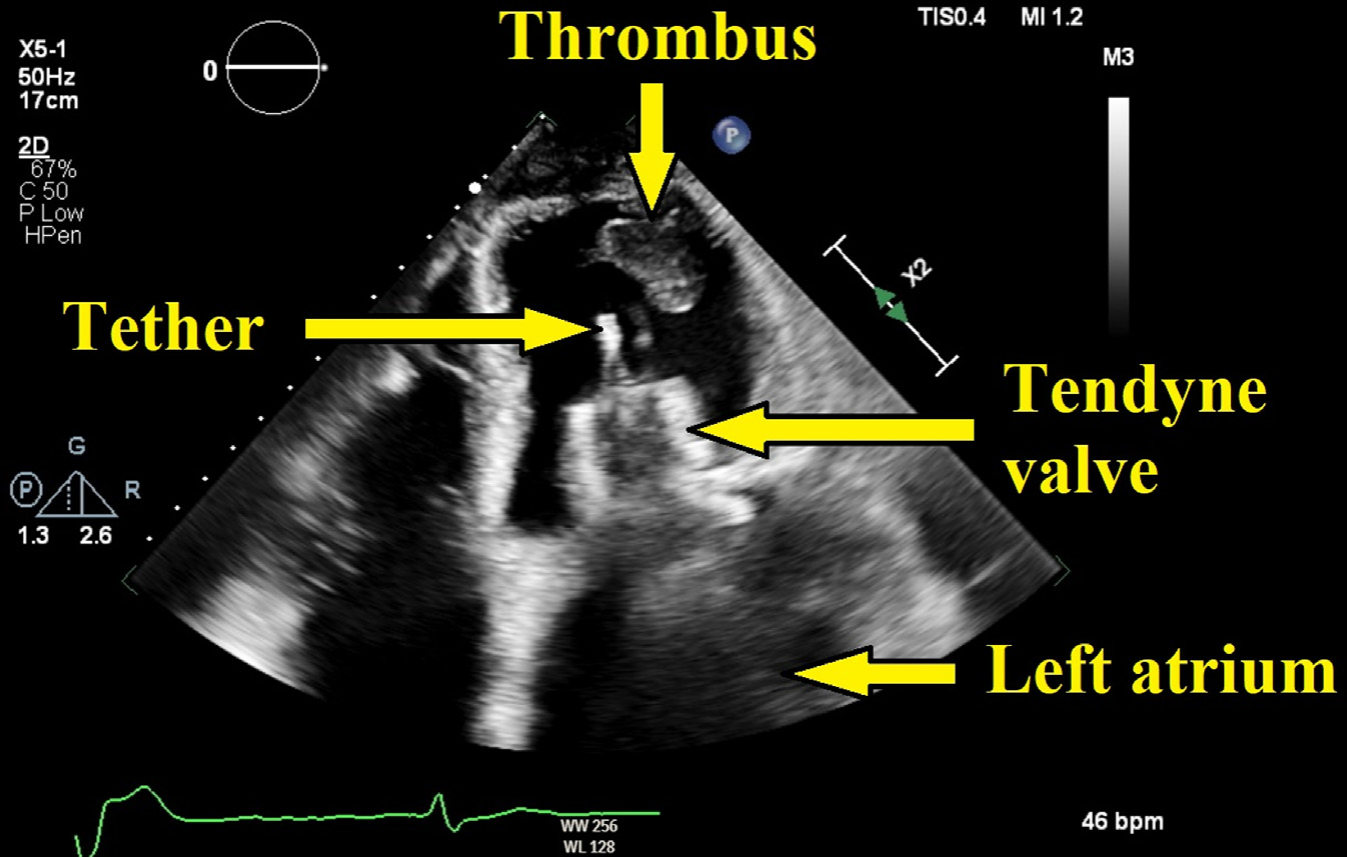
Transthoracic Echocardiogram in Apical 4-Chamber View Transthoracic echocardiogram in apical 4-chamber view (still image) showing the dual-frame transcatheter mitral valve replacement valve and its tether. Note the very large pedunculated mass attached to the apical end of the tether.

His course was later complicated by *Staphylococcus haemolyticus* bacteremia due to an infected peripherally inserted central catheter line, which was removed, and he was treated with a 14-day course of linezolid 600 mg twice daily, followed by cefadroxil 1000 mg twice daily for 4 weeks. He presented again with worsening dyspnea and NYHA functional class IV symptoms approximately 9 months postoperatively. A TTE showed a very large (3.5 × 2.3 cm), pedunculated, and highly mobile mass attached to the apical end of the tether (Figure 3). Left ventricular ejection fraction was 37%. The dual-frame TMVR device was well seated with no transvalvular or paravalvular regurgitation. The transcatheter mitral gradient was 4 mm Hg at a heart rate of 58 beats/min, and pressure half-time was 111 milliseconds (Videos 1 and 2). A transesophageal echocardiogram confirmed normal TMVR device function with no leaflet vegetations (Videos 3 and 4). His blood cultures again grew *S haemolyticus*, leading to the conclusion that the patient had an infected thrombus of the TMVR device tether.

## Outcome and Follow-Up

The patient was deemed at prohibitive risk for surgical intervention. He declined long-term intravenous antibiotics and was treated with oral linezolid followed by indefinite suppressive therapy with cefadroxil 500 mg twice daily. Warfarin was continued.

## Discussion

**Figure 4:**
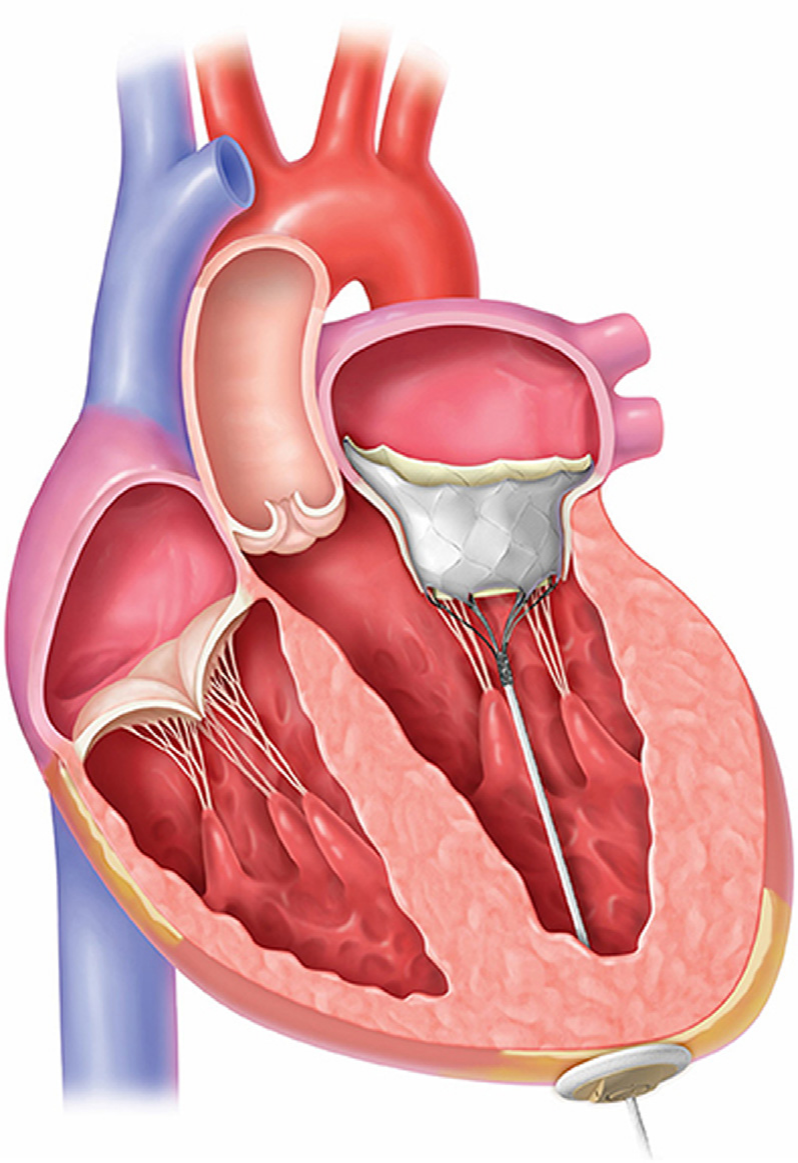
Dual-Frame Transcatheter Mitral Valve Replacement Valve System Reproduced with permission from Abbott, 2024. All rights reserved. Tendyne is a trademark of Abbott or its related companies. Tendyne is approved for use in Europe, the Middle East, and Africa only.

The dual-frame TMVR system, as the name suggests, features a double-frame design (Figure 4). The inner frame supports a trileaflet porcine pericardial valve, whereas the outer frame is contoured to fit the native mitral anatomy while avoiding left ventricular outflow tract obstruction or paravalvular leak. The prosthesis is connected to a braided fiber tether made of high molecular weight polyethylene fastened to an apical pad on the epicardium.

The first implant of this dual-frame TMVR device was performed in acute studies in 2013, followed by compassionate use in 2014, and an early feasibility study case in 2014.^3^ In 2015, the dual-frame TMVR device was acquired by Abbott Laboratories, who then began a pivotal clinical trial of the device in the United States (the SUMMIT trial) in 2018 (NCT03433274). This dual-frame TMVR system became the first TMVR to receive Conformité Européenne mark approval in 2020, followed by U.S. Food and Drug Administration breakthrough device designation in 2021.

The incidence of prosthetic valve endocarditis (PVE) has increased over the past 2 decades, possibly due to improvement in diagnostic modalities and the increasing number of invasive procedures. Causative organisms vary depending on the timing of PVE development postoperatively and the procedural approach. For instance, Enterococci are most common with transfemoral access due to the proximity to genitourinary and intestinal systems, whereas Staphylococci are dominant with the transapical approach.^4^ Compared with aortic prosthetic valves, those in the mitral position are larger, contain more synthetic material, and have slower flow velocity, especially in the presence of atrial fibrillation and a large left atrium. These factors lead to stagnation of flow and increase the risk for thrombosis and endocarditis.

In the Initial Feasibility Study,^5^ the incidence of PVE among 100 patients was 5% (2 cases occurred early postoperatively, and 3 cases occurred 1 to 2 years postoperatively). One patient died from endocarditis on day 29 postoperatively, whereas another patient presented 52 days postoperatively with a mass on the prosthesis (undetermined if vegetation or thrombus) and died from heart failure. One case required surgical removal and replacement of the prosthesis, whereas in the remaining 2 cases, vegetations healed after appropriate antibiotic therapy. Thrombosis occurred in 6% of patients. Leaflet involvement was seen in 4 patients, with the tether in 1 patient, and the cuff in 1 patient. Importantly, these cases occurred early when postoperative medical therapy consisted solely of antiplatelet therapy (aspirin 81 to 325 mg daily). After a protocol change implementing mandatory use of vitamin K antagonist (VKA) for at least 3 months after the procedure (target INR 2.5 to 3.5), no further instances of valve thrombosis were observed throughout 2-year follow-up.^1^^,^^5^

In the European TENDER (The TENDyne European expeRience registry) study,^6^ 108 patients underwent implantation of a dual-frame TMVR. No cases of PVE were reported. Valve thrombosis occurred in only 1 patient 5 days postoperatively. The patient was initially treated with direct oral anticoagulant, which was then transitioned to warfarin, resulting in the subsequent resolution of the thrombus.

Moreover, in a systematic review including 2 prospective studies, 5 retrospective studies, and 19 case reports from across the United States and Europe, data from 319 patients who underwent implantation of a dual-frame TMVR were reported.^7^ The incidence of endocarditis was 2.1%, whereas the incidence of valve thrombosis was 1.9%. Of note, all cases of valve thrombosis were included from the Initial Feasibility Study,^5^ whereas the rest of the included studies and reports were conducted afterward, likely implementing a prophylactic postoperative anticoagulation protocol. Therefore, the incidence of thrombosis associated with the dual-frame TMVR in patients who had appropriate and uninterrupted postoperative anticoagulation is nearly 0%.

However, such a massive vegetation as reported in the current case is, in our experience, unusual, and its clinical course is unpredictable. Considering the difficulties in precisely differentiating a thrombus from endocarditis, such cases should be treated with a combination of microbiologic culture–guided antibiotic therapy and anticoagulation. Depending on the size and physiological consequences, surgical extraction of infected cardiac thrombi may be needed. Although there are reports of successful debulking of large intracardiac vegetations using transcatheter aspiration, this patient did not have a safe approach to perform such a procedure due to the location of the vegetation.

Antibiotic prophylaxis is recommended for all patients with prosthetic valves undergoing dental procedures or other procedures involving incision/biopsy of respiratory mucosa, genitourinary/gastrointestinal procedures in the presence of an infection, and procedures on infected skin or skin structures. However, maintaining good oral health is much more important for the prevention of PVE than antibiotic prophylaxis. Additionally, oral anticoagulation with a VKA is crucial in the first few months after TMVR. Typically, prosthetic valve thrombosis is treated with anticoagulation using VKA with a target INR of 2.5 to 3.5 for at least 6 months. There are very limited data on the use of direct oral anticoagulants for post-TMVR thromboprophylaxis, and their use is currently not recommended or approved.^3^ In patients with prohibitive bleeding risk, an antiplatelet-only strategy may be inevitable. These patients require strict clinical and imaging follow-up to exclude the occurrence of valve thrombosis.^8^

## Conclusions

We present a unique case of a large, infected thrombus associated with the dual-frame TMVR system despite anticoagulation with VKA and appropriate treatment of bacteremia. This case highlights the importance of routine clinical and imaging surveillance of patients with TMVR and discusses the challenges in the postoperative management of these patients.

## Funding Support and Author Disclosures

The authors have reported that they have no relationships relevant to the contents of this paper to disclose.

## Appendix

Video 1 Transthoracic echocardiogram in zoomed-in apical 4-chamber view showing massive and highly mobile vegetation attached to the apical end of the tether.

Video 2 Transthoracic echocardiogram in zoomed-out apical 4-chamber view with color Doppler redemonstrating the vegetation. Note the extremely enlarged gargantuan left atrium (left atrium volume index, 330 mL/m^2^).

Video 3 Transthoracic echocardiogram in midesophageal commissural view showing well-seated and normally functioning dual-frame transcatheter mitral valve replacement valve.

Video 4 Transthoracic echocardiogram in midesophageal commissural view with color Doppler showing no significant paravalvular or transvalvular leak and no leaflet vegetations.

## References

[1] Muller D.W.M., Sorajja P., Duncan A. (2021). 2-year outcomes of transcatheter mitral valve replacement in patients with severe symptomatic mitral regurgitation. J Am Coll Cardiol.

[2] Ascione G., Denti P. (2021). Transcatheter mitral valve replacement and thrombosis: a review. Front Cardiovasc Med.

[3] Dahle G. (2020). Current devices in TMVI and their limitations: focus on Tendyne. Front Cardiovasc Med.

[4] Ivanovic B., Trifunovic D., Matic S. (2019). Prosthetic valve endocarditis - A trouble or a challenge?. J Cardiol.

[5] Sorajja P., Moat N., Badhwar V. (2019). Initial feasibility study of a new transcatheter mitral prosthesis: the first 100 patients. J Am Coll Cardiol.

[6] Wild M.G., Kreidel F., Hell M.M. (2022). Transapical mitral valve implantation for treatment of symptomatic mitral valve disease: a real-world multicentre experience. Eur J Heart Fail.

[7] Ahmed A., Aziz T.A.A., AlAsaad M.M.R. (2023). Transcatheter mitral valve implantation with Tendyne system ten years since the first in-human implant a systematic review. J Cardiothorac Surg.

[8] Pagnesi M., Moroni F., Beneduce A. (2019). Thrombotic risk and antithrombotic strategies after transcatheter mitral valve replacement. JACC Cardiovasc Interv.

